# Palmitoylation reprograms the intestinal mucosal barrier microenvironment: potential mechanisms and promising therapeutic targets

**DOI:** 10.3389/fphar.2026.1905017

**Published:** 2026-09-17

**Authors:** Yanjie Chen, Jinyin Xiao, Huiyuan Liu, Zhenquan Wang

**Affiliations:** 1 Department of Anorectal, The Second Affiliated Hospital of Hunan University of Traditional Chinese Medicine, Changsha, China; 2 Graduate School of Hunan University of Chinese Medicine, Changsha, China

**Keywords:** chemical barrier, immunological barrier, mechanical barrier, mucosal barrier, palmitoylation

## Abstract

Homeostasis of the intestinal mucosal barrier microenvironment is contingent upon the coordinated interplay among mechanical, chemical, and immunological barriers. Recent studies have demonstrated that protein S-palmitoylation, a dynamic and reversible lipid modification, holistically reprograms the intestinal mucosal barrier microenvironment. This is achieved by modulating the membrane localization of tight junction proteins (Claudins and ZO proteins) and E-cadherin/catenin complexes, the secretion and anchoring of MUC2, as well as the membrane recruitment and signaling activation of receptors such as TLR4, NOD2, and NLRP3. Dysregulation of this modification not only drives barrier collapse and chronic inflammation in inflammatory bowel diseases (IBD), such as ulcerative colitis, but also exacerbates pathological progression by modulating the phenotypic remodeling of innate immune cells, including macrophages and neutrophils. Targeting the palmitoylation cycle—including ZDHHC acyltransferases, depalmitoylases (APTs and PPTs), or upstream metabolic enzymes like FASN—using either the broad-spectrum inhibitor 2-BP or selective inhibitors (ML348 and Palmostatin B), has shown promise in preclinical models restore barrier function and attenuate intestinal inflammation. However, it is important to emphasize that the therapeutic potential of these agents in IBD is currently supported primarily by *in vitro* and animal studies, and their clinical efficacy and safety remain to be established. This review systematically proposes a conceptual framework for the “reprogramming of the intestinal mucosal barrier microenvironment by S-palmitoylation.” By integrating current evidence spanning from molecular modifications to barrier functions and from mechanistic insights to therapeutic strategies, we aim to provide novel molecular targets and a theoretical foundation for the development of mucosal healing therapies that are independent of traditional immunosuppression.

## 1 Introduction

The intestinal mucosal barrier represents the most expansive and intricate host-environment interface in the mammalian body. It is composed of a simple columnar epithelium, intercellular tight junctions, an overlying mucus layer, and an underlying network of immune cells within the lamina propria, which collectively constitute a sophisticated “microenvironmental ecosystem” (Allaire et al., 2018a; Peterson and Artis, 2014). Under physiological conditions, this microenvironment strictly maintains three core functions: selective permeability, microbial spatial segregation, and immunosurveillance (Martens et al., 2018). However, when this homeostatic balance is disrupted—as exemplified in inflammatory bowel diseases (IBD) such as ulcerative colitis and Crohn’s disease (Selvakumar et al., 2025)——the loss of barrier integrity is not merely an incipient event; rather, it serves as a central pathological engine driving chronic and relapsing mucosal inflammation (Pastorelli et al., 2013). Therefore, elucidating the regulatory mechanisms of the intestinal mucosal barrier microenvironment and the processes leading to its collapse is a critical prerequisite for the development of next-generation mucosal healing therapies.

Recent groundbreaking studies have begun to delineate the preliminary landscape of how S-palmitoylation reprograms the intestinal mucosal barrier microenvironment. Regarding the mechanical barrier, the palmitoylation-dependent modification of Occludin and Claudins provides the essential structural scaffold required for barrier stability and integrity (Van Itallie et al., 2005). At the level of the chemical barrier, the proper secretion and anchoring of the mucin MUC2 are likewise dependent on the lipidation of its core protein (Pelaseyed et al., 2014). At the level of the immune microenvironment, the S-palmitoylation status of innate immune receptors, such as NLRP3 and TLR4, dictates the sensing thresholds of epithelial and immune cells for microbial signals (Zheng et al., 2023; Płóciennikowska et al., 2015). Furthermore, the sustained activation of pivotal signaling hubs, such as STAT3, has been demonstrated to be contingent upon S-palmitoylation as a mandatory prerequisite (Zhang et al., 2020). Collectively, these disparate lines of evidence converge on a compelling hypothesis: S-palmitoylation does not merely modulate isolated targets, but rather synergistically reprograms the “cell-protein-lipid” interactome that constitutes the barrier microenvironment (Chamberlain and Shipston, 2015), thereby holistically governing the fate of the mucosal barrier.

However, research in this field remains fragmented to date. There is a lack of an integrated perspective that conceptualizes S-palmitoylation as a unified reprogramming engine, capable of systematically orchestrating the intestinal epithelium, the mucus layer, immune sensors, and subepithelial crosstalk to reshape the entire mucosal barrier microenvironment. Crucially, the translational roadmap for converting these mechanistic insights into specific therapeutic interventions targeting the S-palmitoylation cycle remains insufficiently delineated. This review seeks to bridge this knowledge gap by providing a systematic synthesis and outlook. We introduce a novel conceptual framework termed “Palmitoylation-driven Reprogramming of the Intestinal Mucosal Barrier Microenvironment.” By systematically dissecting the core regulatory roles of S-palmitoylation across the mechanical, chemical, and immunological barrier components, we aim to establish this modification as a new paradigm for barrier orchestration. Ultimately, this work seeks to provide a comprehensive molecular blueprint for next-generation mucosal repair therapies that operate independently of traditional immunosuppression.

## 2 Overview of protein palmitoylation

### 2.1 S - Biochemical properties and reversibility of palmitoylation

The defining hallmark that distinguishes S-palmitoylation from other protein lipid modifications lies in the chemical nature of its thioester bond. This process involves the covalent attachment of a saturated 16-carbon palmitate moiety to the sulfhydryl group of a cysteine residue via a labile thioester linkage. This dynamic modification stands in sharp contrast to N-myristoylation, which is anchored by stable amide bonds, and prenylation, which is characterized by permanent thioether bonds (Linder and Deschenes, 2007). The S-palmitoylation cycle is governed by two opposing enzymatic processes: the transfer of the palmitate moiety from palmitoyl-CoA to protein substrates is catalyzed by the ZDHHC family of palmitoyl acyltransferases (PATs), whereas depalmitoylation is mediated by acyl-protein thioesterases. The latter, including cytosolic APT1/APT2 and ABHD17 family members, as well as lysosomal PPT1, are responsible for hydrolyzing the thioester bond and liberating free palmitic acid (De and Sadhukhan, 2018; Lin and Conibear, 2015). The interplay between the expression abundance, subcellular localization, and substrate selectivity of these two enzyme systems collectively dictates the distinct palmitoylation turnover kinetics characteristic of each protein. Taking Ras proteins as a paradigm, Rocks et al. elegantly demonstrated that a continuous palmitoylation-depalmitoylation cycle drives the rapid redistribution of this GTPase between the Golgi apparatus and the plasma membrane. With a half-life as short as tens of minutes, this dynamic cycling is indispensable for maintaining the spatial fidelity of Ras signaling (Rocks et al., 2005).

In contrast to the kinase-phosphatase paradigms of phosphorylation, the S-palmitoylation cycle is uniquely distinguished by its capacity to govern protein function without altering the electrostatic state. Instead, it fundamentally reconfigures a protein’s membrane affinity and subcellular compartmentalization (Salaun et al., 2010; Smotrys and Linder, 2004). The covalent attachment of a lipid anchor confers transient membrane-binding capacity to otherwise hydrophilic cytosolic proteins. Conversely, depalmitoylation facilitates their rapid dissociation back into the cytoplasm or enables their sorting to subsequent destinations via vesicular trafficking (Chamberlain and Shipston, 2015). Arguably, the dynamic reversibility of S-palmitoylation represents an exquisite regulatory equilibrium. Rather than permanently dictating protein fate, it functions as a transient gate that enables the rapid rewiring of signaling networks on a timescale of seconds to minutes (Salaun et al., 2010). Consequently, any aberrant expression or dysregulated activity of the enzymatic machinery governing this cycle can lead to protein mislocalization, which rapidly propagates into cellular dysfunction. This principle provides a compelling rational basis for investigating the homeostatic imbalance of the intestinal mucosal barrier microenvironment.

### 2.2 The functions of DHHC family PATs and de-palmitoylases

The dynamic turnover of protein S-palmitoylation is precisely orchestrated by two functionally opposing enzymatic machineries: PATs, which mediate the covalent attachment of palmitate to substrates, and depalmitoylases, which catalyze the hydrolysis of the resulting thioester bonds (ukata and Fukata, 2010; Greaves and Chamberlain, 2011). In mammals, the PATs catalyzing this reaction are encoded by the ZDHHC gene family, which is characterized by a highly conserved Asp-His-His-Cys (DHHC) tetrapeptide motif. This DHHC module, situated within a cytosolic catalytic pocket, directly participates in the acyl-transfer reaction (Fukata et al., 2004). A total of 23 ZDHHC family members have been identified in the human genome, most of which function as multi-pass transmembrane proteins. These enzymes are distributed across specific endomembrane compartments—including the endoplasmic reticulum (ER), Golgi apparatus, plasma membrane, and endosomes—where their distinct subcellular localizations directly dictate their unique substrate profiles (Ohno et al., 2006). For instance, ZDHHC3 and ZDHHC7 predominantly reside within the Golgi apparatus, where they are responsible for processing proteins traversing the secretory pathway (Fernández-Hernando et al., 2006). In contrast, ZDHHC5 and ZDHHC20 are localized to the plasma membrane, where they modulate the lipidation status of various receptors and signaling molecules (Ohno et al., 2012).

The reversibility of protein palmitoylation is similarly governed by multiple enzymatic systems. To date, the identified depalmitoylases primarily consist of the cytosolic acyl-protein thioesterases APT1 and APT2, members of the ABHD17 family, and the lysosome-localized palmitoyl-protein thioesterase PPT1 (Won and Martin, 2018). APT1 and APT2 represent the most well-characterized depalmitoylases to date. Their catalytic activity relies on a conserved α/β hydrolase fold, which enables the efficient removal of palmitate from a broad spectrum of substrates, including H-Ras, Gα subunits, and various synaptic proteins (Vartak et al., 2014). The ABHD17 family has gained significant attention for its specific regulation of N-Ras palmitoylation turnover. Depletion of ABHD17 markedly decelerates the depalmitoylation kinetics of N-Ras and disrupts its canonical cycling between the plasma membrane and the Golgi apparatus (Won et al., 2018). Despite its sequestration within the lysosomal lumen, emerging evidence suggests that PPT1 can mediate the depalmitoylation of certain cytosolic proteins targeted to the lysosome via autophagic pathways. These findings imply that PPT1 may participate in a broader spectrum of substrate degradation and cellular recycling processes (Lu et al., 1996; Hellsten et al., 1996).

The palmitoylation-depalmitoylation cycle does not represent a mere antagonism between two opposing enzyme systems, but rather constitutes a highly coordinated dynamic equilibrium (Figure 1). Taking Ras proteins as a paradigm, C-terminal farnesylation necessitates subsequent palmitoylation by ZDHHC9 in the Golgi apparatus to confer robust membrane affinity and facilitate plasma membrane targeting (Swarthout et al., 2005). Conversely, APT1 localized at the plasma membrane continuously hydrolyzes the thioester bonds, prompting Ras to return to the Golgi and thereby establishing a coordinated Golgi-plasma membrane shuttling cycle (Ahearn et al., 2011). In highly polarized cells such as intestinal epithelial cells, the discrete subcellular compartmentalization of ZDHHCs and depalmitoylases governs the precise localization of tight junction proteins and mucin-secretion-related SNAREs to the apical or basolateral membranes (Salaun et al., 2010). Any perturbation of this enzymatic equilibrium by inflammatory cytokines or oxidative stress leads to protein mislocalization, which rapidly translates into epithelial barrier dysfunction.

**FIGURE 1:**
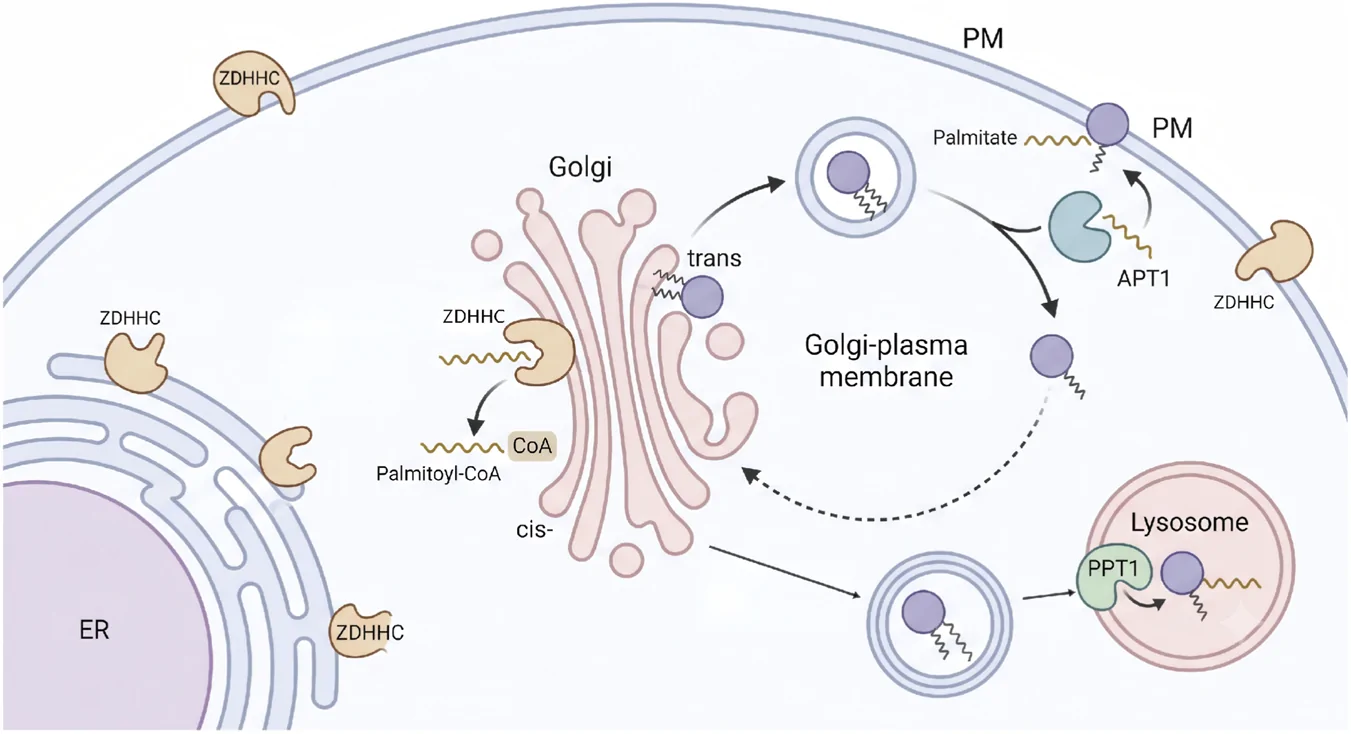
S-palmitoylation modification mechanism diagram. Palmitoyl-CoA is transferred to the cysteine residue of substrate proteins by ZDHHC family acyltransferases, forming a reversible thioester bond, while depalmitoylases (APTs/PPTs) hydrolyze the thioester bond to release free palmitate, thereby dynamically regulating protein membrane affinity, subcellular trafficking, and functional compartmentalization.

### 2.3 Translocation from cytosol to membrane microdomains

S-palmitoylation-mediated subcellular translocation represents a targeted sorting process that directs soluble proteins to specific membrane microdomains. Utilizing quantitative mass spectrometry and phase-separation models, Levental (Levental et al., 2010) demonstrated that palmitoylation levels are significantly higher in raft-associated proteins compared to non-raft counterparts. Furthermore, they showed that the introduction of mutations abolishing palmitoylation leads to the exclusion of these proteins from the raft domains. For example, the Src-family kinase Lck initially associates weakly with the membrane via N-myristoylation, followed by palmitoylation within its unique domain to securely anchor it into lipid rafts of the plasma membrane. Both modifications are indispensable for its proper localization (Yurchak and Sefton, 1995). Thus, palmitoylation serves not as a standalone membrane anchor, but rather as part of a sequential synergy involving multiple lipid modifications. This process converts reversible membrane association into stable, functional residence, thereby enforcing apical-basal directional cues for protein sorting in polarized cells.

## 3 Intestinal mucosal mechanical barrier

### 3.1 Palmitoylation of claudins

Tight junctions (TJs) are the central components of the intestinal epithelial mechanical barrier. They consist of transmembrane proteins, including the claudin family, occludin, and junctional adhesion molecules (JAMs), which are anchored to the actin cytoskeleton via intracellular scaffold proteins to form continuous sealing strands (Zihni et al., 2016). Claudin family members, the structural backbone determining the charge selectivity and pore-based permeability of TJs, are extensively regulated by palmitoylation. Van Itallie (Van Itallie et al., 2005) analyzed multiple claudin isoforms, including Claudin-3, -4, and -5, and identified that palmitoylation is a prevalent modification within their juxtamembrane regions. Notably, mutations at these palmitoylation sites resulted in a diffuse distribution of claudins within intracellular membrane compartments and a failure to efficiently target TJs. Notably, the dependence on palmitoylation varies among different claudin isoforms, a phenomenon that correlates with the length of their cytoplasmic tails and the lipid-contact area of the adjacent transmembrane helices. Furthermore, in epithelial ovarian cancer models, Meng Yuan (Yuan et al., 2020) confirmed that ZDHHC12 directly mediates the palmitoylation of Claudin-3. This modification subsequently suppresses ovarian cancer tumorigenesis by significantly impairing the membrane localization and protein stability of Claudin-3. Although these findings were derived from tumor cells, the underlying mechanism—palmitoylation-mediated protection of claudins against unwarranted proteolysis—is directly extrapolatable to the maintenance of the intestinal epithelial barrier.

### 3.2 The role of the ZO family in assembly connection

Zonula occludens (ZO) proteins are members of the membrane-associated guanylate kinase (MAGUK) family and serve as core scaffold components of the tight junction cytoplasmic plaque (Willott et al., 1993). In mammals, all three members-ZO-1, ZO-2, and ZO-3-possess conserved PDZ, SH3, and GuK domains (Hartsock and Nelson, 2008). Through these modules, they directly interact with the C-terminal sequences of transmembrane proteins, including Claudins, Occludin, and JAM (González-Mariscal et al., 2003). Simultaneously, they cross-link with F-actin via their C-terminal regions, thereby anchoring tight junction strands to the perijunctional actin ring (Fanning and Anderson, 2009). This bridging function establishes ZO proteins as molecular hubs for the mechanical continuity of the epithelial barrier (Van Itallie et al., 2009). Although ZO-1-deficient epithelial cells can still form discontinuous claudin strands, these fragments fail to undergo orderly fusion into continuous sealing fibrils (Umeda et al., 2006; Kuo et al., 2021). This structural defect ultimately results in a decrease in transepithelial electrical resistance (TEER) and a significant increase in the permeability of macromolecular tracers.

However, it is important to note that the aforementioned observations regarding ZO protein function were derived predominantly from epithelial cell lines and non-intestinal model systems. To date, direct experimental evidence demonstrating that ZDHHC-mediated S-palmitoylation of ZO proteins occurs in intestinal epithelial cells or is required for intestinal barrier integrity *in vivo* remains limited. Whether the palmitoylation status of ZO-1, ZO-2, or ZO-3 is dynamically regulated in the context of intestinal inflammation, and whether such regulation contributes to barrier dysfunction in IBD, represent important unanswered questions that warrant future investigation using intestinal-specific models.

### 3.3 Spatial regulation of e-cadherin and catenins

Adherens junctions (AJs) represent the secondary physical linkage between intestinal epithelial cells, subsequent only to tight junctions. The core of this junctional complex is composed of classical Type I E-cadherin and intracellular catenins (Groschwitz and Hogan, 2009). The extracellular domain of E-cadherin mediates homophilic trans-dimerization in a calcium-dependent manner. Meanwhile, its cytoplasmic tail is dynamically coupled to the actin cytoskeleton via β-catenin and α-catenin, thereby integrating the cytoskeletal networks of adjacent cells into a cohesive unit (Harris and Tepass, 2010). This adhesion complex serves not only as a mechanical scaffold for the epithelial barrier but also as an essential prerequisite for the proper assembly of TJs. The formation of AJs triggers the spatial program of epithelial polarization, which subsequently facilitates the orderly recruitment of ZO proteins and Claudins to the apical domain relative to the AJs (Campbell et al., 2017). Consequently, the localization precision of the E-cadherin/catenin complex directly dictates the longitudinal spatial organization of the entire junctional assembly.

Palmitoylation plays a critical but under-recognized role in the spatial regulation of E-cadherin. Studies conducted in non-intestinal epithelial cell models suggests that ZDHHC5-mediated palmitoylation of E-cadherin at the plasma membrane prevents its endocytosis or degradation, thereby ensuring the structural integrity of AJs (Woodley and Collins, 2019). ZDHHC5 is localized to the basolateral membrane and the Golgi apparatus; the E-cadherin palmitoylation it catalyzes occurs during the post-ER trafficking stage. Although palmitoylation-deficient E-cadherin can still correctly fold and reach the plasma membrane via the quality control system, its residence half-life at adherens junctions is significantly shortened (Woodley and Collins, 2019). Concurrently, Ozkan (Ozkan et al., 2023) demonstrated through comparative experiments that ZDHHC5 serves as a pivotal palmitoyltransferase not only for PCDH7 but also for classical cadherins (such as E-cadherin) and various members of the protocadherin family. These findings demonstrate that E-cadherin is a core constituent of the ZDHHC5 enzymatic substrate repertoire. Nevertheless, direct evidence confirming that ZDHHC5-mediated E-cadherin palmitoylation operates in intestinal epithelial cells *in vivo*, or that its dysregulation contributes to the breakdown of adherens junctions in IBD, has not yet been established.

Furthermore, the palmitoylation of catenins also participates in the dynamic regulation of AJs (Figure 2). p120-catenin is a member of the Armadillo family that binds directly to the juxtamembrane domain of E-cadherin. It stabilizes the junctional complex by masking endocytic signal motifs, thereby preventing the constitutive internalization of E-cadherin (Nanes et al., 2012). In an endothelial barrier model, Zebda (Zebda et al., 2013) demonstrated that palmitoylation stabilizes the adherens junction complex, enabling p120-catenin to recruit p190RhoGAP via its C-terminal domain. This recruitment subsequently inhibits RhoA activity, thereby maintaining the low permeability of endothelial and epithelial barriers. While this study was conducted using endothelial barrier models, the functional conservation of p120-catenin across all classical cadherin complexes suggests that its palmitoylation-dependent spatial regulation mechanism is likely generalizable to intestinal epithelial AJs, although this hypothesis awaits direct experimental validation in intestinal-specific models.

**FIGURE 2:**
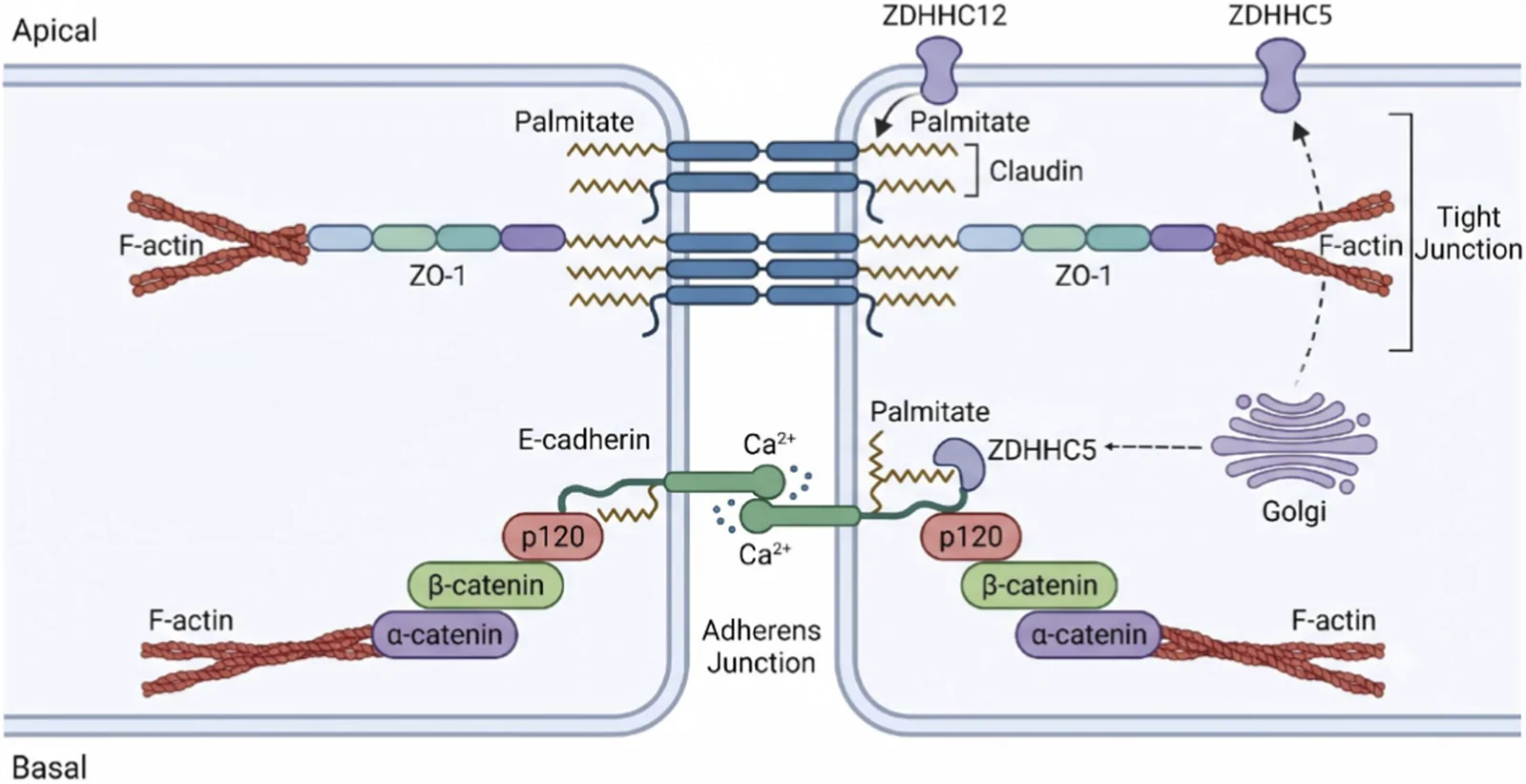
The mechanism of S-palmitoylation in the intestinal mucosal mechanical barrier. S-palmitoylation sustains the intestinal mechanical barrier by facilitating membrane localization of claudins and stabilizing E-cadherin/catenin complexes, thereby ensuring tight-junction integrity and epithelial cohesion.

## 4 Chemical barrier of the intestinal mucosa

### 4.1 Palmitoylation-driven dynamic secretion of mucins

The chemical barrier of the intestinal mucosa is primarily composed of the mucin MUC2, which is synthesized and secreted by goblet cells. This mucin forms a stratified mucus network that overlays the epithelial surface, providing physical segregation between the commensal microbiota and the underlying epithelium (Johansson et al., 2008; Hansson, 2019). Goblet cell homeostasis is characterized not only by a steady rate of mucin secretion but, more importantly, by the cellular tolerance to persistent endoplasmic reticulum (ER) stress induced by the formation of massive intracellular MUC2 aggregates (Allaire et al., 2018b). The secretion of MUC2 is not a continuous, constant leak; rather, it is a process of vesicular exocytosis that is precisely regulated by physiological and immunological cues (Allaire et al., 2018b). Newly synthesized MUC2 undergoes dimerization in the ER and is subsequently translocated to the Golgi apparatus (Birchenough et al., 2016). Through its unique Ca^2+^- and pH-dependent conformational transitions, MUC2 facilitates a dynamic transition from tight intragranular packing to rapid extracellular expansion into a gel-like matrix, thereby establishing a critical physical defense line that prevents direct microbial contact with the epithelium (Ambort et al., 2012). Given that the ER-to-Golgi trafficking of MUC2 dimers represents the critical rate-limiting step in the assembly of the mucus barrier, investigating the potential role of palmitoylation in modulating this transport process is of significant importance. Key evidence derived from intestinal epithelial and goblet cell models indicates that the functional efficacy of MUC2 is critically dependent on its palmitoylation within goblet cells (Johansson et al., 2013). Using a colon epithelial cell line and mouse models, Wei X (Wei et al., 2012) demonstrated that Fatty acid synthase (FASN) mediates the palmitoylation of MUC2 by supplying lipid substrates; this modification serves as a prerequisite for proper MUC2 secretion and the assembly of a dense mucus barrier. Impairment of this modification leads to the collapse of the intestinal chemical barrier, allowing commensal bacteria to contact and activate the mucosal immune system, which subsequently initiates or exacerbates intestinal inflammation. These findings provide direct evidence that MUC2 palmitoylation is a physiologically relevant mechanism governing intestinal chemical barrier function. Furthermore, Ashokkumar Dilly (Dilly et al., 2020) revealed that palmitoylation is a central mechanism driving the malignant phenotype of mucinous adenocarcinoma (MAC). This modification not only establishes a physical defense barrier for the tumor by promoting MUC2 secretion but also suggests that targeting MUC2 palmitoylation could serve as a therapeutic strategy for mucinous colorectal and appendiceal cancers. In summary, MUC2 palmitoylation serves as a critical biochemical axis bridging goblet cell metabolic homeostasis and intestinal mechanical barrier function. By modulating intracellular mucin trafficking and secretion, this modification not only maintains mucosal immune balance but also provides vital pathophysiological insights and molecular targets for the treatment of IBD and MAC.

### 4.2 The gut microbiota drives the remodeling of the mucosal barrier through palmitoylation modification

The intestinal biological barrier is a dynamic network interwoven by the mucus layer, epithelial TJs, and innate immune effector molecules. Through continuous remodeling, this network finely delineates the ecological niches of diverse microbial communities (Okumura and Takeda, 2018). Emerging evidence highlights that gut microbiota and their metabolites orchestrate the remodeling of mucosal barrier functions by modulating the S-palmitoylation of host proteins. Specifically, ZDHHC5-mediated palmitoylation of NOD1/2 is indispensable for their translocation to bacteria-containing endosomes and the subsequent initiation of antibacterial signaling (Lu et al., 2019), a process frequently impaired in Crohn’s disease-associated NOD2 variants. Conversely, the ABHD17 family has been identified as the primary depalmitoylases for NOD2; pharmacological inhibition of ABHD17 enhances the membrane association of NOD2 and restores NF-κB signaling, offering a promising therapeutic strategy for functional rescue of IBD-related variants (Dixon et al., 2025). Beyond innate immunity, microbial metabolites, particularly from *Bacteroides*, influence intestinal permeability by modulating cysteine palmitoylation through 3-ketosphinganine levels (Zhang et al., 2025). Furthermore, dietary long-chain fatty acids (LCFAs) internalised via CD36 can trigger ZDHHC7-catalyzed STAT3 palmitoylation at Cys108, promoting its phosphorylation and driving Th17-mediated inflammatory responses, which ultimately compromises barrier integrity (Wei et al., 2024). Collectively, these findings delineate a pivotal regulatory axis “microbial signaling–palmitoylation modification–barrier remodeling”, providing a novel conceptual framework for understanding IBD pathogenesis and developing targeted interventions.

## 5 Intestinal mucosal immune barrier

### 5.1 Membrane recruitment of pattern recognition receptors

The capacity of the intestinal mucosal immune barrier to discriminate between commensal microbiota and pathogens is highly contingent upon the precise spatial distribution of pattern recognition receptors (PRRs) within specific membrane microdomains. This membrane recruitment process is, to a large extent, programmed by S-palmitoylation (Chen et al., 2025). The membrane trafficking and spatial organization of TLR4 are subject to multiple layers of regulation, among which S-palmitoylation has been proposed as a potential contributing mechanism. Studies in immune cell lines and macrophages have shown that the trafficking of TLR4 from the Golgi apparatus to the plasma membrane, as well as its subsequent residency in lipid rafts, may be influenced by the palmitoylation of juxtamembrane cysteine residues in certain related TLR family members, although direct evidence specifically for TLR4 remains limited (Strassheim et al., 2004). It has been demonstrated that non-palmitoylated TLR4 in some experimental systems is sequestered within the Golgi and fails to respond to LPS stimulation in certain cell types (Strassheim et al., 2004). Upon reaching the plasma membrane, the palmitoyl moieties are thought to insert into the liquid-ordered environment of lipid rafts, thereby potentially establishing a spatially enriched signaling platform for the recruitment of adapter proteins, including MyD88 and TRIF (Płóciennikowska et al., 2015). In the gut microenvironment of IBD, the loss of H2R-mediated inhibitory function abolishes the negative regulation of innate immunity, leaving the TLR4 signaling pathway in a susceptible state. This pro-inflammatory activity of TLR4 has been suggested to be highly dependent on S-palmitoylation-programmed membrane localization. Together, these factors constitute a pathological cascade of “disinhibition, modification-driven activation, and signal amplification” (Smolinska et al., 2016). However, it is important to note that the evidence linking TLR4 palmitoylation directly to its membrane trafficking and signaling activation is largely derived from studies on related TLR family members or from experimental systems overexpressing TLR4 constructs. Direct biochemical evidence demonstrating endogenous TLR4 palmitoylation in intestinal epithelial cells or mucosal macrophages, or showing that this specific modification is strictly required for TLR4 membrane trafficking and signaling in the intestinal microenvironment, remains sparse. Furthermore, the palmitoyl acyltransferases responsible for TLR4 palmitoylation have not been definitively identified in intestinal contexts.

Furthermore, S-palmitoylation (primarily mediated by ZDHHC5) serves as a key molecular switch that drives the recruitment of NOD2 from the cytosol to the plasma membrane. This spatial localization process dictates the functional integrity of NOD2 in sensing bacterial ligands and maintaining intestinal immune homeostasis (Varfolomeev et al., 2025). It’s worth noting that the regulation of NLRP3 by S-palmitoylation is notably complex and context-dependent. Zheng S demonstrated that ZDHHC5-mediated palmitoylation of NLRP3 at Cys837/C838 in the LRR domain promotes NLRP3-NEK7 interaction, inflammasome assembly, and subsequent caspase-1 activation (Zheng et al., 2023), while ZDHHC1-mediated modification at Cys130 and Cys958 directs NLRP3 membrane trafficking to facilitate inflammasome assembly (Nie et al., 2024). ZDHHC7 maintains NLRP3 in the trans-Golgi network under basal conditions and catalyzes Cys126 palmitoylation critical for activation (Yu et al., 2024), whereas ZDHHC12-mediated palmitoylation at Cys844 promotes NLRP3 degradation via chaperone-mediated autophagy, serving as a negative feedback mechanism (Wang et al., 2023). This multi-level regulatory network highlights the dependence of NLRP3 palmitoylation in different situations. Taken together, S-palmitoylation functions as a key molecular switch that maintains intestinal immune homeostasis and drives the pathological cascades of IBD by precisely programming the spatial distribution and molecular interactions of core PRRs, including TLR4, NOD2, and NLRP3.

### 5.2 Phenotypic remodeling of innate immune cells

#### 5.2.1 Macrophage

Intestinal resident macrophages play a pivotal role in maintaining the balance between mucosal immune tolerance and the initiation of host defense responses (Bain and Mowat, 2014). These cells produce negligible amounts of pro-inflammatory cytokines in response to TLR/NOD ligands derived from commensal bacteria, such as LPS and muramyl dipeptide (MDP), yet they retain robust phagocytic and bactericidal capabilities (De Schepper et al., 2018). This functional polarization is highly dependent on the precise organization of cell surface receptors and signaling proteins within specific membrane microdomains, with S-palmitoylation serving as the core mechanism orchestrating this spatial arrangement (Balasubramanian et al., 2024). Using macrophage cell lines and murine colitis models, Jing Lu (Lu et al., 2025) demonstrated that the gut-derived metabolite indole-3-propionic acid (IPA) precisely regulates the palmitoylation levels of key proteins in macrophages by targeting the depalmitoylase PPT1, thereby activating autophagic flux and maintaining macrophage homeostasis. Furthermore, in studies employing intestinal macrophages isolated from murine colitis models, Yuta Hatori (Hatori, 2019) identified that the S-palmitoylation of pro-inflammatory proteins, such as gp91phox, in intestinal macrophages is a critical biochemical mechanism driving ROS production, which subsequently leads to intracellular glutathione (GSH) depletion and redox imbalance. From a therapeutic perspective, using *in vitro* and *in vivo* models of intestinal inflammation, David Meriwether (Meriwether et al., 2019) utilized ApoA-I mimetics to inhibit the S-palmitoylation of COX-2 in intestinal macrophages, which effectively abrogated the production of pro-inflammatory mediators and alleviated intestinal inflammatory injury. Similarly, in cellular and animal models of colitis, Domínguez-Perles (Domínguez-Perles et al., 2025) demonstrated that the palmitoyl modification of epigallocatechin gallate (PEGCG) significantly enhances its immunosuppressive effects. Collectively, these studies provide direct evidence that S-palmitoylation regulates macrophage function in the intestinal microenvironment and represents a therapeutically targetable pathway in IBD. This is achieved by increasing its affinity for the lipid membranes of intestinal macrophages and improving its cellular uptake, thereby more effectively inhibiting TLR4-mediated pro-inflammatory signaling and modulating macrophage polarization. In summary, S-palmitoylation serves as a key post-translational modification mechanism that precisely regulates the spatial organization and biochemical activity of signaling proteins in intestinal macrophages. It plays a pivotal role in maintaining mucosal immune homeostasis, regulating redox balance, and mediating the resolution of intestinal inflammation.

#### 5.2.2 Neutrophil

During the active phase of ulcerative colitis (UC), a large number of neutrophils migrate from the circulation across the endothelium into the lamina propria and subsequently infiltrate the crypt lumen via transepithelial migration (Mumy and McCormick, 2009). This leads to the formation of crypt abscesses, which represent the most characteristic histopathological hallmark distinguishing UC from Crohn’s disease (Magro et al., 2013). The chemotactic recruitment and transepithelial migration of neutrophils are highly dependent on the S-palmitoylation of signaling molecules, such as Src family kinases (Birukov et al., 2004). By regulating the spatial polarization and dynamic activation of proteins within membrane microdomains, this modification serves as a key biochemical switch that drives neutrophil infiltration into inflamed tissues. Studies have indicated that neutrophils achieve precise regulation of TLR signal transduction through FASN-mediated palmitoylation of MyD88 (Kim et al., 2019). This mechanism illustrates how metabolic flux influences immune signaling pathways. Notably, S-palmitoylation serves as a dynamic hub for the membrane localization of signaling proteins in neutrophils. By mediating the spatial assembly of inflammatory complexes, this modification offers a promising and reversible pharmacological intervention path to inhibit excessive neutrophil recruitment and tissue injury during the active phase of IBD (Li et al., 2026). Despite the central importance of S-palmitoylation in regulating neutrophil activation, the precise dynamic molecular mechanisms by which this modification drives transepithelial infiltration and induces tissue injury within the specific intestinal microenvironment of IBD remain poorly understood, representing a critical knowledge gap.

## 6 The pharmacological mechanism of remodeling the mucosal barrier through palmitoylation

### 6.1 Metabolic pathway regulators

The development and progression of intestinal inflammation are closely associated with the metabolic reprogramming of innate immune cells, such as neutrophils. Using human and murine neutrophils as well as colitis models, Kim YC (Kim et al., 2019) demonstrated that that under inflammatory stimulation, FASN expression is significantly upregulated in neutrophils, which expands the endogenous palmitoyl-CoA pool and directly drives the S-palmitoylation of the key adapter protein MyD88. Acting as a “molecular anchor”, this modification facilitates the dynamic translocation and spatial clustering of MyD88 into lipid raft microdomains of the plasma membrane. This process effectively triggers the TLR signaling pathway and the subsequent NF-κB-mediated release of pro-inflammatory cytokines (Li et al., 2026) (Figure 3). These findings establish a direct causal link between FASN-mediated metabolic flux, MyD88 palmitoylation, and neutrophil-driven intestinal inflammation in IBD-relevant models. Pharmacological intervention studies have confirmed that Orlistat, by targeting and inhibiting FASN-mediated metabolic flux, depletes the substrate pool required for protein modification at its source. This action blocks the S-palmitoylation and subsequent membrane-initiated signaling of key pathogenic proteins such as AKT, thereby achieving therapeutic efficacy in preclinical models of metabolic-related diseases, including hepatocellular carcinoma and IBD (Bu et al., 2024). This finding establishes FASN-mediated S-palmitoylation as a critical hub linking cellular metabolic flux to inflammatory responses. Furthermore, it provides a significant theoretical basis for exploring the potential of restoring intestinal mucosal homeostasis through the pharmacological targeting of metabolic nodes, although clinical validation remains a crucial next step.

**FIGURE 3:**
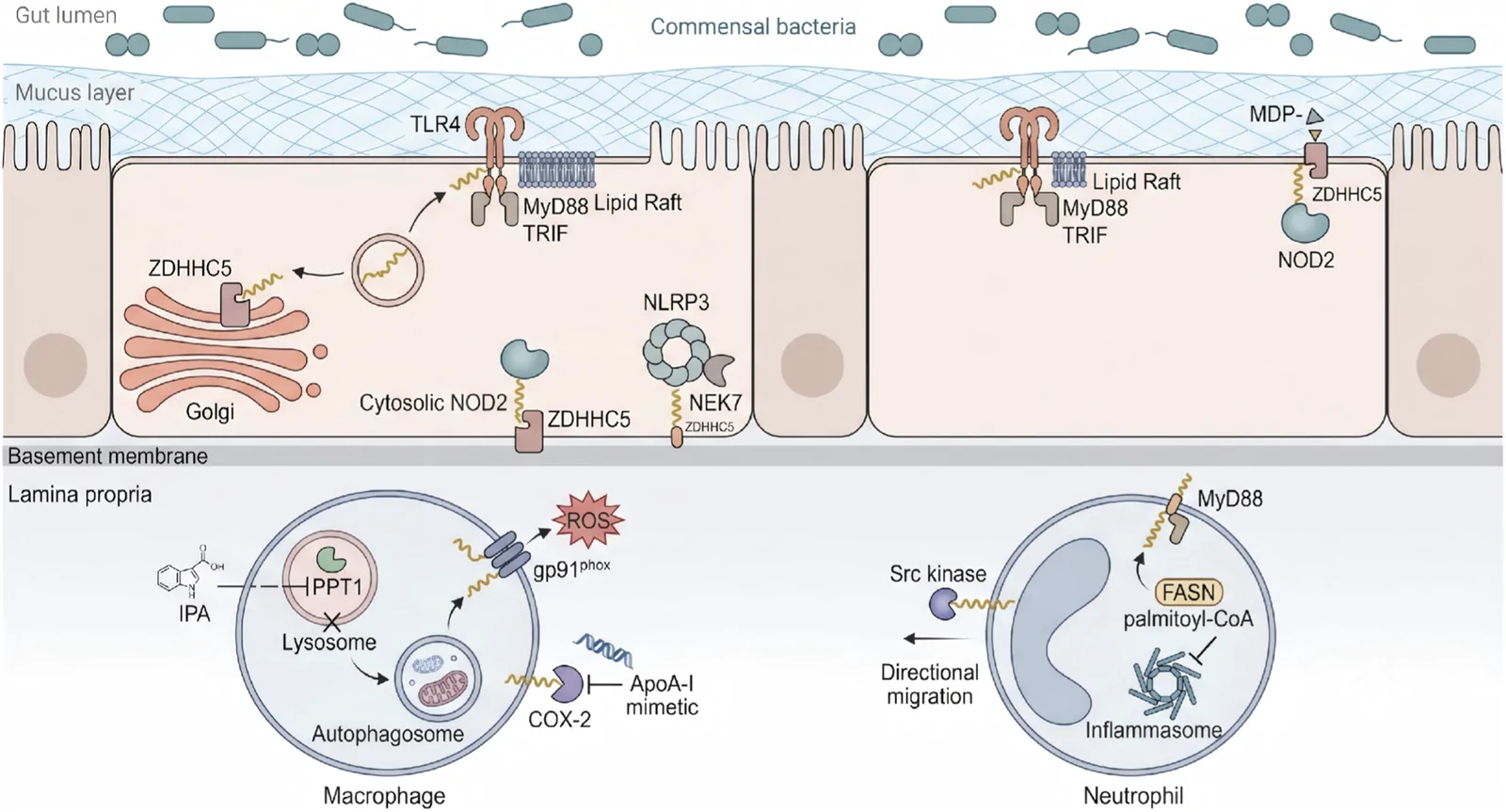
The mechanism of S-palmitoylation in the intestinal mucosal immune barrier. S-palmitoylation governs the intestinal mucosal immune barrier by directing membrane recruitment of pattern-recognition receptors (TLR4, NOD2, and NLRP3) for innate signal activation, and by dynamically remodeling macrophage polarization and neutrophil trafficking through lipid-raft-dependent signaling and metabolic enzymes, thereby determining the balance between immune homeostasis and inflammatory tissue injury.

### 6.2 Broad-spectrum palmitoyl transferase inhibitor

Broad-spectrum palmitoyltransferase inhibitors, typified by 2-bromopalmitate (2-BP), serve as essential pharmacological tools for elucidating the biological functions of protein S-palmitoylation (Cao et al., 2026). These inhibitors primarily function by covalently binding to the catalytic domain (DHHC motif) of the ZDHHC protein family. This action competitively blocks the association of Palmitoyl-CoA with the enzyme, thereby inhibiting the lipid modification of a wide range of substrate proteins (Jennings et al., 2009; Chavda et al., 2014). In murine models of diet-induced colitis, Wei Y (Wei et al., 2024) demonstrated that pharmacological blockade of STAT3 S-palmitoylation by 2-BP effectively disrupts the pathological coupling between CD36-mediated lipid metabolism and Th17 pro-inflammatory responses, thereby significantly alleviating diet-induced intestinal inflammation in these experimental settings. This study provides direct *in vivo* evidence in an intestinal inflammation model that broad-spectrum palmitoylation inhibition can ameliorate mucosal inflammation in mice.

Furthermore, broad-spectrum inhibition strategies in experimental models have confirmed that suppressing palmitoylation significantly reduces the transendothelial migration efficiency of neutrophils. This reduction subsequently mitigates the disruption of the mucosal mechanical barrier caused by inflammatory cell infiltration (Marin et al., 2012). Despite its lack of isoform selectivity, 2-BP has played an indispensable role in validating palmitoylation as a potential therapeutic target for intestinal inflammation. Furthermore, it has laid the pharmacological foundation for the subsequent development of precision drugs targeting specific ZDHHC isoenzymes.

### 6.3 Depalmitoylating enzyme inhibitors

Protein S-palmitoylation is a highly dynamic and reversible post-translational modification, the homeostasis of which is maintained by the coordinated action of palmitoyltransferases (ZDHHCs) and depalmitoylating enzymes (Mesquita et al., 2024). Depalmitoylating enzymes primarily comprise acyl-protein thioesterases (APTs) and palmitoyl-protein thioesterases (PPTs) (Shang et al., 2025). The development of inhibitors targeting these enzymes, such as the broad-spectrum inhibitor Palmostatin B (Lin et al., 2017) and highly selective inhibitors ML348 (Virlogeux et al., 2021) and ML349 (He et al., 2025), has become a key strategy for modulating the functions of signal transduction proteins. Mechanistically, inhibition of depalmitoylating enzymes induces a “hyper-palmitoylated” state of target proteins, thereby disrupting their dynamic cycling between the endomembrane system and the plasma membrane. For instance, pharmacological inhibition of APT-1/2-mediated depalmitoylation induces the spatial mislocalization of oncoproteins such as NRAS. This effect abrogates oncogenic signaling without altering total palmitoylation levels, offering a precise intervention strategy that targets protein dynamic cycling for the treatment of NRAS-mutant melanoma (Vujic et al., 2016). Early studies using intestinal epithelial cell models have shown that Palmostatin B maintains the palmitoylation status of Wnt2B by inhibiting depalmitoylating enzymes, which in turn enhances Wnt signaling-mediated intestinal epithelial regeneration (Klaus et al., 2014). These findings raise the possibility that modulating the homeostasis of protein lipid modifications could serve as a potential therapeutic strategy for promoting intestinal mucosal barrier repair, although this hypothesis remains to be tested *in vivo* models of IBD. In conclusion, by precisely regulating the lipid modification dynamics of key signaling and scaffold proteins, depalmitoylase inhibitors not only modulate tumor signaling pathways but also enhance epithelial regeneration in experimental systems. Thus, they represent a mechanistically intriguing pharmacological strategy for maintaining and restoring intestinal mucosal barrier function that warrants further investigation. Critically, at present, the therapeutic potential of drugs targeting palmitoylation modifications in diseases such as IBD and other intestinal mucosal barrier dysfunction disorders mainly stems from the mechanism exploration conducted *in vitro* and in animal experiments. There is still a lack of systematic pharmacodynamic evaluation and clinical research data to support their clinical application. Therefore, while the available evidence provides a strong mechanistic rationale, all palmitoylation-targeting compounds discussed here should be considered experimental tools with demonstrated preclinical activity rather than established therapies for IBD.

### 6.4 Limitations and translational challenges of palmitoylation-targeting inhibitors

Although inhibitors targeting protein palmitoylation hold significant therapeutic promise for intestinal mucosal barrier repair, their clinical translation is hindered by multifaceted challenges, including poor selectivity, inherent toxicity, and biological complexity. The pan-inhibitor 2-BP exerts its effects via covalent modification of the ZDHHC catalytic domains (Jennings et al., 2009); however, its concurrent inhibition of depalmitoylases (APT1/2) and various lipid metabolic enzymes poses substantial off-target risks and cytotoxicity. Consequently, 2-BP remains a crude pharmacological probe rather than a viable therapeutic lead (Chavda et al., 2014). While the selective APT inhibitors ML348 and ML349 have achieved breakthroughs in isoform specificity, their application is restricted by suboptimal tissue distribution and poor solubility (Vujic et al., 2016). Furthermore, the multi-target inhibitory profile of Palmostatin B against the ABHD17 family has reshaped our understanding of its mechanism of action, serving as a critical reminder that biochemical enzyme selectivity does not necessarily translate into physiological drug selectivity. The development of palmitoylation-targeted therapies has reached a critical juncture; future breakthroughs lie in bridging the chasm between biochemical potency and systemic pharmacological safety, ultimately achieving a synergistic balance between mucosal healing efficacy and minimized off-target toxicity through optimized pharmacokinetics.

## 7 Conclusions and future directions

Protein S-palmitoylation dynamically coordinates the mechanical, chemical, and immunological pillars of the intestinal mucosal barrier to sustain microenvironmental homeostasis. Nevertheless, major gaps persist: the substrate specificity of ZDHHC acyltransferases and depalmitoylases across distinct intestinal cell types (enterocytes, goblet cells, Paneth cells, macrophages, and neutrophils) remains largely uncharacterized, and whether these modifications operate in a cell-type- or context-dependent manner under homeostasis versus inflammation is unclear.

To address these issues, we propose: (1) cell-type-specific conditional knockouts using Vil1-, Muc2-, Lyz1-, or LysM-Cre drivers to dissect causal roles; (2) *in vivo* palmitoylome mapping by proximity-labeling (TurboID/APEX2) combined with acyl-biotin exchange chemistry; (3) intravital two-photon microscopy with palmitoylation-sensitive fluorescent reporters to visualize real-time membrane trafficking during colitis; and (4) patient-derived organoids with CRISPR-Cas9 editing to validate human translational potential. Integrating these genetic, proteomic, and imaging strategies will yield a mechanistic, cell-resolved framework of palmitoylation-driven barrier reprogramming, paving the way for precision therapies that promote mucosal healing independent of broad immunosuppression.

## References

[B1] Ahearn I. M. Haigis K. Bar-Sagi D. Philips M. R. (2011). Regulating the regulator: post-translational modification of RAS. Nat. Rev. Mol. Cell Biol. 13 (1), 39–51. 10.1038/nrm3255 22189424 PMC3879958

[B2] Allaire J. M. Crowley S. M. Law H. T. Chang S. Y. Ko H. J. Vallance B. A. (2018a). The intestinal epithelium: central coordinator of mucosal immunity. Trends Immunol. 39 (9), 677–696. 10.1016/j.it.2018.04.002 29716793

[B3] Allaire J. M. Morampudi V. Crowley S. M. Stahl M. Yu H. Bhullar K. (2018b). Frontline defenders: goblet cell mediators dictate host-microbe interactions in the intestinal tract during health and disease. Am. J. Physiol. Gastrointest. Liver Physiol. 314 (3), G360–G377. 10.1152/ajpgi.00181.2017 29122749 PMC5899238

[B4] Ambort D. Johansson M. E. Gustafsson J. K. Nilsson H. E. Ermund A. Johansson B. R. (2012). Calcium and pH-dependent packing and release of the gel-forming MUC2 mucin. Proc. Natl. Acad. Sci. United States 109 (15), 5645–5650. 10.1073/pnas.1120269109 22451922 PMC3326483

[B5] Bain C. C. Mowat A. M. (2014). Macrophages in intestinal homeostasis and inflammation. Immunol. Rev. 260 (1), 102–117. 10.1111/imr.12192 24942685 PMC4141699

[B6] Balasubramanian A. Hsu A. Y. Ghimire L. Tahir M. Devant P. Fontana P. (2024). The palmitoylation of gasdermin D directs its membrane translocation and pore formation during pyroptosis. Sci. Immunol. 9 (94), eadn1452. 10.1126/sciimmunol.adn1452 38530158 PMC11367861

[B7] Birchenough G. M. Nyström E. E. Johansson M. E. Hansson G. C. (2016). A sentinel goblet cell guards the colonic crypt by triggering Nlrp6-dependent Muc2 secretion. Science 352 (6293), 1535–1542. 10.1126/science.aaf7419 27339979 PMC5148821

[B8] Birukov K. G. Leitinger N. Bochkov V. N. Garcia J. G. N. (2004). Signal transduction pathways activated in human pulmonary endothelial cells by OxPAPC, a bioactive component of oxidized lipoproteins. Microvasc. Res. 67 (1), 18–28. 10.1016/j.mvr.2003.09.004 14709399

[B9] Bu L. Zhang Z. Chen J. Fan Y. Guo J. Su Y. (2024). High-fat diet promotes liver tumorigenesis via palmitoylation and activation of AKT. Gut 73 (7), 1156–1168. 10.1136/gutjnl-2023-330826 38191266

[B10] Campbell H. K. Maiers J. L. DeMali K. A. (2017). Interplay between tight junctions and adherens junctions. Exp. Cell Res. 358 (1), 39–44. 10.1016/j.yexcr.2017.03.061 28372972 PMC5544570

[B11] Cao H. Liu X. Zhu S. Nie H. Liu S. Fan J. (2026). The emerging roles of protein lipidation in fertility and reproductive disorders: mechanisms and therapeutic implications. Biomolecules 16 (3), 389. 10.3390/biom16030389 41897325 PMC13024116

[B12] Chamberlain L. H. Shipston M. J. (2015). The physiology of protein S-acylation. Physiol. Rev. 95 (2), 341–376. 10.1152/physrev.00032.2014 25834228 PMC4551212

[B13] Chavda B. Arnott J. A. Planey S. L. (2014). Targeting protein palmitoylation: selective inhibitors and implications in disease. Expert Opin. Drug Discov. 9 (9), 1005–1019. 10.1517/17460441.2014.933802 24967607

[B14] Chen Y. Yue S. Yu L. Cao J. Liu Y. Deng A. (2025). Regulation and function of the cGAS-STING pathway: mechanisms, post-translational modifications, and therapeutic potential in immunotherapy. Drug Des. Devel Ther. 19, 1721–1739. 10.2147/DDDT.S501773 40098909 PMC11911240

[B15] De I. Sadhukhan S. (2018). Emerging roles of DHHC-mediated protein S-palmitoylation in physiological and pathophysiological context. Eur. J. Cell Biol. 97 (5), 319–338. 10.1016/j.ejcb.2018.03.005 29602512

[B65] De Schepper S. Verheijden S. Aguilera-Lizarraga J. Viola M. F. Boesmans W. Stakenborg N. (2018). Self-maintaining gut macrophages are essential for intestinal homeostasis. Cell 175 (2), 400–415.e13. 10.1016/j.cell.2018.07.048 30173915

[B16] Dilly A. Honick B. D. Lee Y. J. Bartlett D. L. Choudry H. A. (2020). Rational application of targeted therapeutics in mucinous colon/appendix cancers with positive predictive factors. Cancer Med. 9 (5), 1753–1767. 10.1002/cam4.2847 31958897 PMC7050077

[B17] Dixon C. L. Martin N. R. Niphakis M. J. Cravatt B. F. Fairn G. D. (2025). Attenuating ABHD17 isoforms augments the S-acylation and function of NOD2 and a subset of crohn's disease-associated NOD2 variants. Cell Mol. Gastroenterol. Hepatol. 19 (6), 101491. 10.1016/j.jcmgh.2025.101491 40054525 PMC12005342

[B18] Domínguez-Perles R. Medrano-Padial C. García-Viguera C. Medina S. (2025). Palmitic acid esterification boosts epigallocatechin gallate's immunomodulatory effects in intestinal inflammation. Biomolecules 15 (8), 1208. 10.3390/biom15081208 40867652 PMC12384343

[B19] Fanning A. S. Anderson J. M. (2009). Zonula occludens-1 and -2 are cytosolic scaffolds that regulate the assembly of cellular junctions. Ann. N Y Acad. Sci. 1165, 113–120. 10.1111/j.1749-6632.2009.04440.x 19538295 PMC3759978

[B20] Fernández-Hernando C. Fukata M. Bernatchez P. N. Fukata Y. Lin M. I. Bredt D. S. (2006). Identification of Golgi-localized acyl transferases that palmitoylate and regulate endothelial nitric oxide synthase. J. Cell Biol. 174 (3), 369–377. 10.1083/jcb.200601051 16864653 PMC2064233

[B21] Fukata M. Fukata Y. Adesnik H. Nicoll R. A. Bredt D. S. (2004). Identification of PSD-95 palmitoylating enzymes. Neuron 44 (6), 987–996. 10.1016/j.neuron.2004.12.005 15603741

[B22] González-Mariscal L. Betanzos A. Nava P. Jaramillo B. E. (2003). Tight junction proteins. Prog. Biophys. Mol. Biol. 81 (1), 1–44. 10.1016/s0079-6107(02)00037-8 12475568

[B23] Greaves J. Chamberlain L. H. (2011). DHHC palmitoyl transferases: substrate interactions and (patho)physiology. Trends Biochem. Sci. 36 (5), 245–253. 10.1016/j.tibs.2011.01.003 21388813

[B24] Groschwitz K. R. Hogan S. P. (2009). Intestinal barrier function: molecular regulation and disease pathogenesis. J. Allergy Clin. Immunol. 124 (1), 3–20. 10.1016/j.jaci.2009.05.038 19560575 PMC4266989

[B25] Hansson G. C. (2019). Mucus and mucins in diseases of the intestinal and respiratory tracts. J. Intern Med. 285 (5), 479–490. 10.1111/joim.12910 30963635 PMC6497544

[B26] Harris T. J. Tepass U. (2010). Adherens junctions: from molecules to morphogenesis. Nat. Rev. Mol. Cell Biol. 11 (7), 502–514. 10.1038/nrm2927 20571587

[B27] Hartsock A. Nelson W. J. (2008). Adherens and tight junctions: structure, function and connections to the actin cytoskeleton. Biochim. Biophys. Acta 1778 (3), 660–669. 10.1016/j.bbamem.2007.07.012 17854762 PMC2682436

[B28] Hatori Y. (2019). Analyzing the redox status of intracellular glutathione and its application to an intestinal bowel disease model. Yakugaku Zasshi 139 (12), 1523–1530. 10.1248/yakushi.19-00184 31787639

[B29] He Y. Li S. Jiang L. Wu K. Chen S. Su L. (2025). Palmitic acid accelerates endothelial cell injury and cardiovascular dysfunction via palmitoylation of PKM2. Adv. Sci. (Weinh) 12 (5), e2412895. 10.1002/advs.202412895 39665133 PMC11791964

[B30] Hellsten E. Vesa J. Olkkonen V. M. Jalanko A. Peltonen L. (1996). Human palmitoyl protein thioesterase: evidence for lysosomal targeting of the enzyme and disturbed cellular routing in infantile neuronal ceroid lipofuscinosis. EMBO J. 15 (19), 5240–5245. 8895569 PMC452268

[B33] Jennings B. C. Nadolski M. J. Ling Y. Baker M. B. Harrison M. L. Deschenes R. J. (2009). 2-Bromopalmitate and 2-(2-hydroxy-5-nitro-benzylidene)-benzo[b]thiophen-3-one inhibit DHHC-mediated palmitoylation *in vitro* . J. Lipid Res. 50 (2), 233–242. 10.1194/jlr.M800270-JLR200 18827284 PMC2636914

[B34] Johansson M. E. Phillipson M. Petersson J. Velcich A. Holm L. Hansson G. C. (2008). The inner of the two Muc2 mucin-dependent mucus layers in colon is devoid of bacteria. Proc. Natl. Acad. Sci. United States 105 (39), 15064–15069. 10.1073/pnas.0803124105 18806221 PMC2567493

[B35] Johansson M. E. Sjövall H. Hansson G. C. (2013). The gastrointestinal mucus system in health and disease. Nat. Rev. Gastroenterol. Hepatol. 10 (6), 352–361. 10.1038/nrgastro.2013.35 23478383 PMC3758667

[B36] Kim Y. C. Lee S. E. Kim S. K. Jang H. D. Hwang I. Jin S. (2019). Toll-like receptor mediated inflammation requires FASN-dependent MYD88 palmitoylation. Nat. Chem. Biol. 15 (9), 907–916. 10.1038/s41589-019-0344-0 31427815

[B37] Klaus C. Schneider U. Hedberg C. Schütz A. K. Bernhagen J. Waldmann H. (2014). Modulating effects of acyl-CoA synthetase 5-derived mitochondrial Wnt2B palmitoylation on intestinal wnt activity. World J. Gastroenterol. 20 (40), 14855–14864. 10.3748/wjg.v20.i40.14855 25356045 PMC4209548

[B38] Kuo W. T. Zuo L. Odenwald M. A. Madha S. Singh G. Gurniak C. B. (2021). The tight junction protein ZO-1 is dispensable for barrier function but critical for effective mucosal repair. Gastroenterology 161 (6), 1924–1939. 10.1053/j.gastro.2021.08.047 34478742 PMC8605999

[B39] Levental I. Lingwood D. Grzybek M. Coskun U. Simons K. (2010). Palmitoylation regulates raft affinity for the majority of integral raft proteins. Proc. Natl. Acad. Sci. United States 107 (51), 22050–22054. 10.1073/pnas.1016184107 21131568 PMC3009825

[B40] Li S. Dou X. Sun J. Xiao Y. Wang T. Shan Q. (2026). Protein S-palmitoylation: potential strategy for inflammation-related diseases. J. Pharm. Anal. 16 (5), 101490. 10.1016/j.jpha.2025.101490 42206205 PMC13202024

[B41] Lin D. T. Conibear E. (2015). ABHD17 proteins are novel protein depalmitoylases that regulate N-Ras palmitate turnover and subcellular localization. Elife 4, e11306. 10.7554/eLife.11306 26701913 PMC4755737

[B42] Lin D. T. S. Davis N. G. Conibear E. (2017). Targeting the ras palmitoylation/depalmitoylation cycle in cancer. Biochem. Soc. Trans. 45 (4), 913–921. 10.1042/BST20160303 28630138

[B43] Linder M. E. Deschenes R. J. (2007). Palmitoylation: policing protein stability and traffic. Nat. Rev. Mol. Cell Biol. 8 (1), 74–84. 10.1038/nrm2084 17183362

[B44] Lu J. Y. Verkruyse L. A. Hofmann S. L. (1996). Lipid thioesters derived from acylated proteins accumulate in infantile neuronal ceroid lipofuscinosis: correction of the defect in lymphoblasts by recombinant palmitoyl-protein thioesterase. Proc. Natl. Acad. Sci. United States 93 (19), 10046–10050. 10.1073/pnas.93.19.10046 8816748 PMC38333

[B45] Lu Y. Zheng Y. Coyaud É. Zhang C. Selvabaskaran A. Yu Y. (2019). Palmitoylation of NOD1 and NOD2 is required for bacterial sensing. Science 366 (6464), 460–467. 10.1126/science.aau6391 31649195

[B46] Lu J. Wang H. Zhang H. Li J. Li H. Chen Q. (2025). Gut metabolite Indole-3-Propionic acid regulates macrophage autophagy through PPT1 inhibiting aging-related myocardial fibrosis. Adv. Sci. (Weinh) 12 (34), e01070. 10.1002/advs.202501070 40539882 PMC12442707

[B47] Magro F. Langner C. Driessen A. Ensari A. Geboes K. Mantzaris G. J. (2013). European Society of Pathology (ESP); European Crohn's and Colitis Organisation (ECCO). European consensus on the histopathology of inflammatory bowel disease. J. Crohns Colitis 7 (10), 827–851. 10.1016/j.crohns.2013.06.001 23870728

[B48] Marin E. P. Derakhshan B. Lam T. T. Davalos A. Sessa W. C. (2012). Endothelial cell palmitoylproteomic identifies novel lipid-modified targets and potential substrates for protein acyl transferases. Circ. Res. 110 (10), 1336–1344. 10.1161/CIRCRESAHA.112.269514 22496122 PMC3428238

[B49] Martens E. C. Neumann M. Desai M. S. (2018). Interactions of commensal and pathogenic microorganisms with the intestinal mucosal barrier. Nat. Rev. Microbiol. 16 (8), 457–470. 10.1038/s41579-018-0036-x 29904082

[B50] Meriwether D. Sulaiman D. Volpe C. Dorfman A. Grijalva V. Dorreh N. (2019). Apolipoprotein A-I mimetics mitigate intestinal inflammation in COX2-dependent inflammatory bowel disease model. J. Clin. Invest. 129 (9), 3670–3685. 10.1172/JCI123700 31184596 PMC6715371

[B51] Mesquita F. S. Abrami L. Linder M. E. Bamji S. X. Dickinson B. C. van der Goot F. G. (2024). Mechanisms and functions of protein S-acylation. Nat. Rev. Mol. Cell Biol. 25 (6), 488–509. 10.1038/s41580-024-00700-8 38355760 PMC12010433

[B52] Mumy K. L. McCormick B. A. (2009). The role of neutrophils in the event of intestinal inflammation. Curr. Opin. Pharmacol. 9 (6), 697–701. 10.1016/j.coph.2009.10.004 19854677 PMC2798135

[B53] Nanes B. A. Chiasson-MacKenzie C. Lowery A. M. Ishiyama N. Faundez V. Ikura M. (2012). p120-catenin binding masks an endocytic signal conserved in classical cadherins. J. Cell Biol. 199 (2), 365–380. 10.1083/jcb.201205029 23071156 PMC3471230

[B54] Nie L. Fei C. Fan Y. Dang F. Zhao Z. Zhu T. (2024). Consecutive palmitoylation and phosphorylation orchestrates NLRP3 membrane trafficking and inflammasome activation. Mol. Cell 84 (17), 3336–3353.e7. 10.1016/j.molcel.2024.08.001 39173637

[B55] Ohno Y. Kihara A. Sano T. Igarashi Y. (2006). Intracellular localization and tissue-specific distribution of human and yeast DHHC cysteine-rich domain-containing proteins. Biochim. Biophys. Acta 1761 (4), 474–483. 10.1016/j.bbalip.2006.03.010 16647879

[B56] Ohno Y. Kashio A. Ogata R. Ishitomi A. Yamazaki Y. Kihara A. (2012). Analysis of substrate specificity of human DHHC protein acyltransferases using a yeast expression system. Mol. Biol. Cell 23 (23), 4543–4551. 10.1091/mbc.E12-05-0336 23034182 PMC3510016

[B57] Okumura R. Takeda K. (2018). Maintenance of intestinal homeostasis by mucosal barriers. Inflamm. Regen. 38, 5. 10.1186/s41232-018-0063-z 29619131 PMC5879757

[B58] Ozkan N. E. Yigit B. N. Degirmenci B. S. Qureshi M. H. Yapici G. N. Kamacıoglu A. (2023). Cell cycle-dependent palmitoylation of protocadherin 7 by ZDHHC5 promotes successful cytokinesis. J. Cell Sci. 136 (6), jcs260266. 10.1242/jcs.260266 36762613

[B59] Pastorelli L. De Salvo C. Mercado J. R. Vecchi M. Pizarro T. T. (2013). Central role of the gut epithelial barrier in the pathogenesis of chronic intestinal inflammation: lessons learned from animal models and human genetics. Front. Immunol. 4, 280. 10.3389/fimmu.2013.00280 24062746 PMC3775315

[B60] Pelaseyed T. Bergström J. H. Gustafsson J. K. Ermund A. Birchenough G. M. H. Schütte A. (2014). The mucus and mucins of the goblet cells and enterocytes provide the first defense line of the gastrointestinal tract and interact with the immune system. Immunol. Rev. 260 (1), 8–20. 10.1111/imr.12182 24942678 PMC4281373

[B61] Peterson L. W. Artis D. (2014). Intestinal epithelial cells: regulators of barrier function and immune homeostasis. Nat. Rev. Immunol. 14 (3), 141–153. 10.1038/nri3608 24566914

[B62] Płóciennikowska A. Hromada-Judycka A. Borzęcka K. Kwiatkowska K. (2015). Co-operation of TLR4 and raft proteins in LPS-induced pro-inflammatory signaling. Cell Mol. Life Sci. 72 (3), 557–581. 10.1007/s00018-014-1762-5 25332099 PMC4293489

[B63] Rocks O. Peyker A. Kahms M. Verveer P. J. Koerner C. Lumbierres M. (2005). An acylation cycle regulates localization and activity of palmitoylated ras isoforms. Science 307 (5716), 1746–1752. 10.1126/science.1105654 15705808

[B64] Salaun C. Greaves J. Chamberlain L. H. (2010). The intracellular dynamic of protein palmitoylation. J. Cell Biol. 191 (7), 1229–1238. 10.1083/jcb.201008160 21187327 PMC3010063

[B66] Selvakumar B. Samsudin R. (2025). Intestinal barrier dysfunction in inflammatory bowel disease: pathophysiology to precision therapeutics. Inflamm. Bowel Dis. 31 (12), 3450–3464. 10.1093/ibd/izaf225 41189113

[B67] Shang J. Ding M. Zhou X. (2025). Recent advances in S-palmitoylation and its emerging roles in human diseases. J. Hematol. Oncol. 18 (1), 83. 10.1186/s13045-025-01738-7 40890756 PMC12403631

[B68] Smolinska S. Groeger D. Perez N. R. Schiavi E. Ferstl R. Frei R. (2016). Histamine receptor 2 is required to suppress innate immune responses to bacterial ligands in patients with inflammatory bowel disease. Inflamm. Bowel Dis. 22 (7), 1575–1586. 10.1097/MIB.0000000000000825 27271490

[B69] Smotrys J. E. Linder M. E. (2004). Palmitoylation of intracellular signaling proteins: regulation and function. Annu. Rev. Biochem. 73, 559–587. 10.1146/annurev.biochem.73.011303.073954 15189153

[B70] Strassheim D. Asehnoune K. Park J. S. Kim J. Y. He Q. Richter D. (2004). Phosphoinositide 3-kinase and akt occupy central roles in inflammatory responses of toll-like receptor 2-stimulated neutrophils. J. Immunol. 172 (9), 5727–5733. 10.4049/jimmunol.172.9.5727 15100319

[B71] Swarthout J. T. Lobo S. Farh L. Croke M. R. Greentree W. K. Deschenes R. J. (2005). DHHC9 and GCP16 constitute a human protein fatty acyltransferase with specificity for H- and N-Ras. J. Biol. Chem. 280 (35), 31141–31148. 10.1074/jbc.M504113200 16000296

[B72] ukata Y. Fukata M. (2010). Protein palmitoylation in neuronal development and synaptic plasticity. Nat. Rev. Neurosci. 11 (3), 161–175. 10.1038/nrn2788 20168314

[B73] Umeda K. Ikenouchi J. Katahira-Tayama S. Furuse K. Sasaki H. Nakayama M. (2006). ZO-1 and ZO-2 independently determine where claudins are polymerized in tight-junction strand formation. Cell 126 (4), 741–754. 10.1016/j.cell.2006.06.043 16923393

[B31] Van Itallie C. M. Gambling T. M. Carson J. L. Anderson J. M. (2005). Palmitoylation of claudins is required for efficient tight-junction localization. J. Cell Sci. 118 (Pt 7), 1427–1436. 10.1242/jcs.01735 15769849

[B32] Van Itallie C. M. Fanning A. S. Bridges A. Anderson J. M. (2009). ZO-1 stabilizes the tight junction solute barrier through coupling to the perijunctional cytoskeleton. Mol. Biol. Cell 20 (17), 3930–3940. 10.1091/mbc.e09-04-0320 19605556 PMC2735491

[B74] Varfolomeev E. Mohanan V. Vucic D. (2025). S-Palmitoylation regulates signaling mediated by NLRP3 and other innate immune receptors. Mol. Cell 85 (20), 3862–3873. 10.1016/j.molcel.2025.09.009 41106374

[B75] Vartak N. Papke B. Grecco H. E. Rossmannek L. Waldmann H. Hedberg C. (2014). The autodepalmitoylating activity of APT maintains the spatial organization of palmitoylated membrane proteins. Biophys. J. 106 (1), 93–105. 10.1016/j.bpj.2013.11.024 24411241 PMC3907232

[B76] Virlogeux A. Scaramuzzino C. Lenoir S. Carpentier R. Louessard M. Genoux A. (2021). Increasing brain palmitoylation rescues behavior and neuropathology in huntington disease mice. Sci. Adv. 7 (14), eabb0799. 10.1126/sciadv.abb0799 33789888 PMC8011966

[B77] Vujic I. Sanlorenzo M. Esteve-Puig R. Vujic M. Kwong A. Tsumura A. (2016). Acyl protein thioesterase 1 and 2 (APT-1, APT-2) inhibitors palmostatin B, ML348 and ML349 have different effects on NRAS mutant melanoma cells. Oncotarget 7 (6), 7297–7306. 10.18632/oncotarget.6907 26771141 PMC4872786

[B78] Wang L. Cai J. Zhao X. Ma L. Zeng P. Zhou L. (2023). Palmitoylation prevents sustained inflammation by limiting NLRP3 inflammasome activation through chaperone-mediated autophagy. Mol. Cell 83 (2), 281–297.e10. 10.1016/j.molcel.2022.12.002 36586411

[B79] Wei X. Yang Z. Rey F. E. Ridaura V. K. Davidson N. O. Gordon J. I. (2012). Fatty acid synthase modulates intestinal barrier function through palmitoylation of mucin 2. Cell Host Microbe. 11 (2), 140–152. 10.1016/j.chom.2011.12.006 22341463 PMC3285413

[B80] Wei Y. Li J. Li J. Liu C. Guo X. Liu Z. (2024). Dietary long-chain fatty acids promote colitis by regulating palmitoylation of STAT3 through CD36-mediated endocytosis. Cell Death Dis. 15 (1), 60. 10.1038/s41419-024-06456-5 38233383 PMC10794235

[B81] Willott E. Balda M. S. Fanning A. S. Jameson B. Van Itallie C. Anderson J. M. (1993). The tight junction protein ZO-1 is homologous to the drosophila discs-large tumor suppressor protein of septate junctions. Proc. Natl. Acad. Sci. United States 90 (16), 7834–7838. 10.1073/pnas.90.16.7834 8395056 PMC47237

[B82] Won S. J. Martin B. R. (2018). Temporal profiling establishes a dynamic S-Palmitoylation cycle. ACS Chem. Biol. 13 (6), 1560–1568. 10.1021/acschembio.8b00157 29733200 PMC6192522

[B83] Won S. J. Cheung See Kit M. Martin B. R. (2018). Protein depalmitoylases. Crit. Rev. Biochem. Mol. Biol. 53 (1), 83–98. 10.1080/10409238.2017.1409191 29239216 PMC6009847

[B84] Woodley K. T. Collins M. O. (2019). S-acylated Golga7b stabilises DHHC5 at the plasma membrane to regulate cell adhesion. EMBO Rep. 20 (10), e47472. 10.15252/embr.201847472 31402609 PMC6776912

[B85] Yu T. Hou D. Zhao J. Lu X. Greentree W. K. Zhao Q. (2024). NLRP3 Cys126 palmitoylation by ZDHHC7 promotes inflammasome activation. Cell Rep. 43 (4), 114070. 10.1016/j.celrep.2024.114070 38583156 PMC11130711

[B86] Yuan M. Chen X. Sun Y. Jiang L. Xia Z. Ye K. (2020). ZDHHC12-mediated claudin-3 S-palmitoylation determines ovarian cancer progression. Acta Pharm. Sin. B 10 (8), 1426–1439. 10.1016/j.apsb.2020.03.008 32963941 PMC7488353

[B87] Yurchak L. K. Sefton B. M. (1995). Palmitoylation of either Cys-3 or Cys-5 is required for the biological activity of the lck tyrosine protein kinase. Mol. Cell Biol. 15 (12), 6914–6922. 10.1128/MCB.15.12.6914 8524258 PMC230946

[B88] Zebda N. Tian Y. Tian X. Gawlak G. Higginbotham K. Reynolds A. B. (2013). Interaction of p190RhoGAP with C-terminal domain of p120-catenin modulates endothelial cytoskeleton and permeability. J. Biol. Chem. 288 (25), 18290–18299. 10.1074/jbc.M112.432757 23653363 PMC3689971

[B89] Zhang M. Zhou L. Xu Y. Yang M. Xu Y. Komaniecki G. P. (2020). A STAT3 palmitoylation cycle promotes TH17 differentiation and colitis. Nature 586 (7829), 434–439. 10.1038/s41586-020-2799-2 33029007 PMC7874492

[B90] Zhang Y. H. Dai C. S. Wang Y. J. Wang W. Y. Qi T. T. Xia M. C. (2025). Intestinal permeability of N-acetylcysteine is driven by gut microbiota-dependent cysteine palmitoylation. Nat. Commun. 16 (1), 4623. 10.1038/s41467-025-59916-7 40389439 PMC12089494

[B91] Zheng S. Que X. Wang S. Zhou Q. Xing X. Chen L. (2023). ZDHHC5-mediated NLRP3 palmitoylation promotes NLRP3-NEK7 interaction and inflammasome activation. Mol. Cell 83 (24), 4570–4585.e7. 10.1016/j.molcel.2023.11.015 38092000

[B92] Zihni C. Mills C. Matter K. Balda M. S. (2016). Tight junctions: from simple barriers to multifunctional molecular gates. Nat. Rev. Mol. Cell Biol. 17 (9), 564–580. 10.1038/nrm.2016.80 27353478

